# Training Non-Medical Staff for SARS-CoV-2 Swab Collection on a Psychiatric Old Age Ward

**DOI:** 10.1192/bjo.2022.90

**Published:** 2022-06-20

**Authors:** Joanna Legg, Sophie Ouabbou, Susan Hay

**Affiliations:** 1 Royal Free London NHS Foundation Trust, London, United Kingdom; 2 Barnet, Enfield and Haringey NHS Mental Health Trust, London, United Kingdom; 3 Camden and Islington NHS Trust, London, United Kingdom

## Abstract

**Aims:**

In the second half of 2020 patients admitted to Highgate Mental Health Centre had to isolate in their rooms until a negative SARS-CoV-2 test result was obtained. This was stressful for both patients, who were unwell in their mental state, and staff. Swabs for PCR testing were only being collected by junior doctors which meant that out of hours, this responsibility would fall exclusively upon the duty doctor. There were often significant delays to obtain a sample. We decided to train non-medical staff on an old age ward so that the responsibility of collecting samples could be shared with nurses and healthcare assistants.

**Methods:**

In November 2020 we held one training session with several members of staff from our ward. In the following days we did one to one training sessions with the members of staff who, due to their shifts, were not available for the original training session. We excluded admissions that happened prior to SARS-CoV-2 being mandatory, those where the patient refused to be swabbed, and those patients who were transferred from another institution already with a pre-admission swab.

**Results:**

There were 37 admissions, of which we included 30 based on the exclusion criteria. 17 admissions occurred prior to training and 13 after the training sessions. Prior to training, it took 1.059 days to obtain a sample and it took 0.846 days after the training sessions.

**Conclusion:**

Providing a training session to enable nurses and healthcare assistants to take samples for SARS-CoV-2 testing reduced the amount of time between admission and obtaining a swab sample. We therefore shortened the first step of the process that leads to obtaining a negative result and enable a patient to come out of isolation.

